# Attitudes of university students towards mandatory COVID-19 vaccination: a cross-sectional survey: Antonio Sciurti

**DOI:** 10.1093/eurpub/ckac131.344

**Published:** 2022-10-25

**Authors:** A Sciurti, V Baccolini, E Renzi, G Migliara, C De Vito, C Marzuillo, P Villari

**Affiliations:** Department of Public Health and Infectious Diseases, Sapienza University of Rome, Rome, Italy

## Abstract

**Background:**

Vaccination is an effective public health measure to control the COVID-19 pandemic. In Italy, vaccination against SARS-CoV-2 was made compulsory by law for some job categories, such as healthcare and education workers. Although students required a valid COVID-19 certificate to access university, they were never subjected to mandatory vaccination. In this context, we investigated their attitudes towards mandating COVID-19 vaccination to be able to access schools and universities.

**Methods:**

We conducted a cross-sectional survey from September 2021 to February 2022. A multivariable logistic regression model was built to identify predictors of positive attitudes towards the outcome. Adjusted odds ratio (aOR) and 95% confidence intervals (CIs) were calculated.

**Results:**

We collected 5287 questionnaires, grouped into three periods (September-October 2021, November-December 2021 and January-February 2022). The highest proportion of students supporting COVID-19 mandatory vaccination (62.5%) was found between November and December 2021. Multivariable analysis showed that November-December and January-February participants had higher odds of having a positive attitude towards the vaccine mandate than September-October respondents (aOR=1.26, 95% CI:1.09-1-48; and aOR=1.22, 95% CI:1.01-1.48). Other positive predictors were age (aOR=1.01, 95% CI:1.01-1.03), higher levels of perceived COVID-19 severity (aOR=1.09, 95% CI:1.05-1.14), concern for the emergency (aOR=1.09, 95% CI:1.05-1.14), getting vaccinated for fear of infecting other people or being infected (aOR=1.08, 95% CI:1.04-1.12; and aOR=1.07, 95% CI:1.03-1.10) and believing that vaccines could end the pandemic (aOR=1.49, 95% CI:1.41-1.56).

**Conclusions:**

Attitudes towards COVID-19 mandatory vaccination changed over time, probably in relation to the pandemic trends. Moreover, feeling involved in the pandemic situation seems to be related with a positive attitude.

**Key messages:**

• Pandemic trends may affect the attitude towards vaccine mandates. Mandating vaccines to access universities could be taken into account in emergency situations to ensure a safer learning environment.

• Involvement in the pandemic situation seems related with a supportive attitude towards vaccine mandates, suggesting that awareness is a key factor to be addressed to implement mandatory vaccination.

